# Efficient Deep Learning Architecture for Detection and Recognition of Thyroid Nodules

**DOI:** 10.1155/2020/1242781

**Published:** 2020-07-29

**Authors:** Jingzhe Ma, Shaobo Duan, Ye Zhang, Jing Wang, Zongmin Wang, Runzhi Li, Yongli Li, Lianzhong Zhang, Huimin Ma

**Affiliations:** ^1^Cooperative Innovation Center of Internet Healthcare, Zhengzhou University, Zhengzhou 450000, China; ^2^School of Information Engineering, Zhengzhou University, Zhengzhou 450000, China; ^3^Department of Health Management, Henan Provincial People's Hospital, Zhengzhou 450003, China

## Abstract

Ultrasonography is widely used in the clinical diagnosis of thyroid nodules. Ultrasound images of thyroid nodules have different appearances, interior features, and blurred borders that are difficult for a physician to diagnose into malignant or benign types merely through visual recognition. The development of artificial intelligence, especially deep learning, has led to great advances in the field of medical image diagnosis. However, there are some challenges to achieve precision and efficiency in the recognition of thyroid nodules. In this work, we propose a deep learning architecture, you only look once v3 dense multireceptive fields convolutional neural network (YOLOv3-DMRF), based on YOLOv3. It comprises a DMRF-CNN and multiscale detection layers. In DMRF-CNN, we integrate dilated convolution with different dilation rates to continue passing the edge and the texture features to deeper layers. Two different scale detection layers are deployed to recognize the different sizes of the thyroid nodules. We used two datasets to train and evaluate the YOLOv3-DMRF during the experiments. One dataset includes 699 original ultrasound images of thyroid nodules collected from a local health physical center. We obtained 10,485 images after data augmentation. Another dataset is an open-access dataset that includes ultrasound images of 111 malignant and 41 benign thyroid nodules. Average precision (AP) and mean average precision (mAP) are used as the metrics for quantitative and qualitative evaluations. We compared the proposed YOLOv3-DMRF with some state-of-the-art deep learning networks. The experimental results show that YOLOv3-DMRF outperforms others on mAP and detection time on both the datasets. Specifically, the values of mAP and detection time were 90.05 and 95.23% and 3.7 and 2.2 s, respectively, on the two test datasets. Experimental results demonstrate that the proposed YOLOv3-DMRF is efficient for detection and recognition of thyroid nodules for ultrasound images.

## 1. Introduction

With its ever-increasing incidence, the thyroid nodule is one of the most common nodular tumors in the adult population [1, 2]. The timely diagnosis of thyroid nodules is extremely essential. Ultrasonography is the primary and preferred screening method for the clinical diagnosis of thyroid nodules. The diagnosis comprises a fine needle aspiration biopsy (FNAB) and a follow-up treatment [3]. Clinically, doctors typically diagnose thyroid nodules by experience. However, this method could result in an ambiguous diagnosis [4], thereby causing excessive treatments such as unnecessary biopsy and surgery. With an increase in the number of patients, the radiologists are subjected to increased workloads. This may cause reduced average diagnostic time on each case, thereby leading to an increased incidence of misdiagnosis [5]. It is critical to accurately detect and recognize thyroid nodules as benign or malignant.

Traditionally, thyroid nodules have been mainly diagnosed visually, through human observation. However, it is difficult to accurately judge complicated thyroid nodules in ultrasound images through this method. Good old-fashioned artificial intelligence (GOFAI) and handcrafted features method [6] were developed to address the aforementioned problem. However, the two main drawbacks of GOFAI and the handcrafted features method are their high time complexity and unsatisfactory universality. The development of artificial intelligence, especially deep learning, has brought excellent advances in the field of medical image diagnosis. However, there are some challenges to achieve precision and efficiency in the recognition of thyroid nodules.

We propose a deep learning architecture, you only look once v3 dense multireceptive fields convolutional neural network (YOLOv3-DMRF), based on YOLOv3. It comprises a dense multireceptive fields convolutional neural network (DMRF-CNN) and multiscale detection layers. In DMRF-CNN, we integrate dilated convolution with different dilation rates to continue passing the edge and the texture features to deeper layers. Two different scale detection layers are deployed to recognize the different sizes of the thyroid nodules.

**Figure 1:**
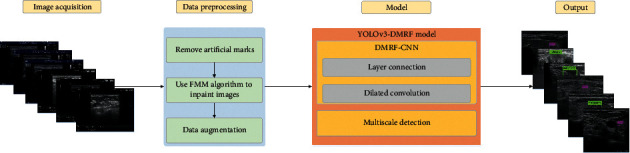
Frame diagram for recognition of thyroid nodules.

Figure 1 illustrates the frame diagram for the recognition of thyroid nodules. We use the ultrasound images of thyroid nodules as our dataset in the first stage. Subsequently, we process the original data through several operations that include the removal of artificial marks, image inpainting, and data augmentation. Lastly, the architecture YOLOv3-DMRF is presented to complete the detection and recognition.

## 2. Related Work

In this section, we discuss some related works that focus on ultrasound images of thyroid nodules. They mainly comprise three stages: GOFAI, handcrafted features method [6], and deep learning method.

Initially, medical image analysis was performed with GOFAI or an expert system, which was similar to a rule-based image processing system. This method analyzed medical images by using low-level pixel processing and mathematical modeling method to solve tasks. Low-level pixel processing mainly included the following filters: edge detector, region growing, and line detector [7, 8]. Mathematical modeling mainly included fitting lines, circles, and ellipses [9]. However, the GOFAI approach was often brittle, and it required massive manual intervention.

Furthermore, several researchers used the handcrafted features method. For example, Toki and Tanaka [10] used the scale-invariant feature transform (SIFT) [11] to extract features in images to identify prostate cancer, whereas Niwas et al. [12] used the least squares support vector machine (LS-SVM) to diagnose breast cancer based on the texture characteristics of biopsy data. Furthermore, Basavanhally et al. [13] proposed a new multiview classifier on different sizes to identify the essential features of an image. These methods are based on the handcrafted features of pathological images of breast cancer. Nevertheless, the high variability of ultrasound images of thyroid nodules is a challenge in recognizing the benign or malignant types of the nodules. Moreover, in the case of changes in the characteristics such as distortion, clipping, lighting, and damage, the performances of these algorithms would worsen. Therefore, the universality on these previous methods is not stable.

Regarding the deep learning methods, the convolutional neural network (CNN) [14] was applied in image analysis, e.g., LeNet [15], AlexNet [16], visual geometry group network (VGGNet) [17], GoogLeNet [18], and residual network (ResNet) [19]. The CNN architecture can automatically extract the multilevel features. However, CNN for image classification progressively reduces resolution, which may further reduce the detailed spatial information. Dilated filters were developed in the à trous algorithm for efficient wavelet decomposition in [20], and they have been used in image pixel prediction to facilitate efficient computation [21, 22]. Comparison of the traditional convolutional kernel with the dilated convolution kernel of the same size shows that the latter requires a lesser number of network parameters, and it expands the receptive fields of the kernels to a greater degree to obtain almost the same information. Models based on dilated convolution have been actively explored for semantic segmentation of medical images. For example, Moeskops et al. [23] used dilated convolutions to segment images of brain MRI. The results showed improved segmentation performance while using the dilated convolutions procedure for segmentation of two different sets of images. Additionally, CNN has shown rapid development in image recognition, e.g., region-based CNN (R-CNN) [24], single shot detector (SSD) [25], Fast-RCNN [26], and Faster-RCNN [27]. However, these methods have high detection and recognition times. YOLO [28–30] is one of the state-of-the-art object detection systems designed by Joseph Redmon and Ali Farhadi. Compared to other object detection systems, the most outstanding feature of YOLO is high efficiency. YOLOv3, which is the third version, shows improved accuracy in addition to high efficiency. Therefore, YOLOv3 is used in our experiments.

Furthermore, some deep learning methods have been used in ultrasound images of thyroid nodules. For example, Chi et al. [31] used the fine-tuned GoogLeNet model to achieve good results on the open-access thyroid ultrasound image database [32]. Chi's method detected the location of thyroid nodules by manually gauging the position of the nodule, but failed to do so automatically. Li et al. [33] and Wang et al. [34] proposed an improved Fast R-CNN model for the detection of papillary thyroid carcinoma. Song et al. [35] proposed a multitask cascade CNN model by using SSD framework and a spatial pyramid network to detect thyroid nodules coarsely and finely, respectively. The aforementioned deep learning model can recognize thyroid nodules with a guaranteed satisfactory performance. However, the problems of complex network architectures with several parameters and large detection time cost must be solved urgently.

To address these problems, we present a deep learning architecture, YOLOv3-DMRF, based on YOLOv3, to detect and recognize thyroid nodules automatically and efficiently.

## 3. Data Preprocessing

### 3.1. Removal of Artificial Marks and Image Inpainting

**Figure 2:**
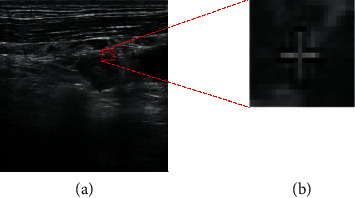
Ultrasound images of thyroid nodules with unclear artificial marks: (a) original image of having unclear artificial marks; (b) artificial marks are mixed with background pixels.

Pathologists mark the outline of thyroid nodules during clinical diagnosis. This becomes a double-edged sword for the following reason. Although we can obtain the position and the size of nodules easily, additional noise is introduced during detection and recognition. Moreover, as shown in Figure 2, artificial marks merge into background pixels.

**Figure 3:**
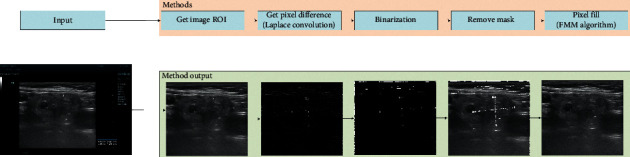
Ultrasonic image removal method and output.

It is necessary to remove artificial marks in the preprocessing stage on the dataset. Based on numerous experiments, we use the Laplacian convolution [36] and the fast marching method (FMM) algorithm [37] to locate the positions of the artificial marks and remove them.  Figure 3 illustrates the pipeline diagram of the processing. Firstly, the region of interest (ROI) of the ultrasound image is obtained. Subsequently, we use the Laplacian operator to find the locations of the artificial marks in the image. Next, we convert the input image into a binary one by using a reasonable threshold to obtain the artificial marks. Furthermore, we find the position of the marks in the original image based on the binary image and remove the marks. Finally, we perform the ultrasound image inpainting by using the INPAINT_TELEA algorithm of OpenCV [38].

### 3.2. Image Augmentation

To avoid overfitting, we use certain augmentation methods such as color jitter, change saturation, exposure, and hue to produce ultrasonic images as a supplement. It should be noted that we abandon the random transformation of angles because the aspect rate of thyroid nodules affects the discrimination between benign and malignant. In the experiments, we set jitter to 0.3, which represents that the ultrasonic images are randomly cropped and flipped using rates from 0 to 0.3. Meanwhile, we set both saturation and exposure to 1.5. The hue is 0.1, which represents the random generation of pictures in the range of −0.1 ∼ 0.1 hues.

## 4. YOLOv3-DMRF Model

**Figure 4:**
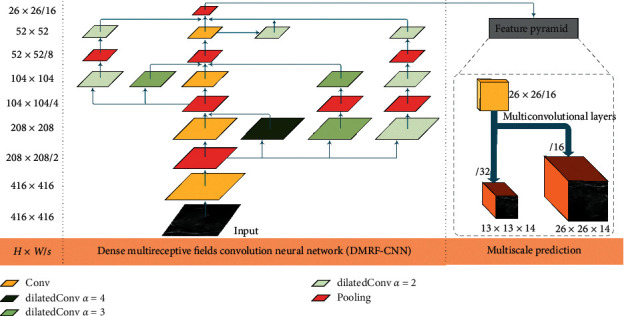
YOLOv3-DMRF architecture for thyroid nodules recognition.

In this work, we propose a deep learning architecture, YOLOv3-DMRF, for detection and recognition of thyroid nodules, as shown in Figure 4. It can be used in auxiliary diagnoses. Its depth is 81, and H and W denote the height and width of the feature maps, respectively. *s* (*s* = 1, 2, ..., 32) denotes the down-sampling rate the for the input images (416 × 416). Different colors represent feature maps with different operations. *α* denotes the dilation rate.

### 4.1. IoU for Anchor Boxes

Object detection must choose anchor boxes in the training stage. In this work, we use the K-means algorithm to obtain original anchor boxes based on the training datasets that are derived from the original thyroid ultrasound images.  Algorithm 1 outlines the procedure of the same. First, we randomly select K coordinates from the ground truth boxes set (gtbox) as the coordinates of the initial cluster centers: *C*_k_, where *k* ∈ {1, 2, ..., K}. Furthermore, for the coordinates of the ground truth boxes, we calculate the distance to the K cluster centers and assign the coordinates to the set of the nearest center. This set is denoted as *C*set_k_. Next, we calculate the mean of all the coordinates in cluster Cset_k_ to update the coordinates of cluster center *C*_k_. Finally, for the coordinates of all K cluster centers, we repeat the above steps until the coordinates of cluster center *C*_k_ do not change. The coordinates of the K cluster centers are the coordinates of the anchor boxes.

We use equation (1) to compute the distance between the K cluster centers for each of the other boxes:

(1) $$\begin{array}{c} d\left(b_{1},b_{2}\right)=1-IoU\left(b_{1},b_{2}\right). \end{array}$$

Intersection over union (IoU) is a measure of the distance for two crossing objects, and it is defined as follows:

(2) $$\begin{array}{c} IoU\left(b_{1},b_{2}\right)=\frac{\min\left(w_{1},w_{2}\right).\min\left(h_{1},h_{2}\right)}{w_{1}h_{1}+w_{2}h_{2}-\min\left(w_{1},w_{2}\right).\min\left(h_{1},h_{2}\right)}. \end{array}$$

For each object *b*_i_, *w*_i_ and *h*_i_ are the width and the height, respectively. When the sizes of both the objects, *b*_1_ and *b*_2_, are equal, IoU reaches its maximum value of 1.

Additionally, to evaluate the effect of anchor box by the K-means algorithm, we calculate the average IoU (Avg IoU), which is defined as follows:

(3) $$\begin{array}{c} \text{AvgIoU}=\frac{1}{n}\sum_{p=1}^{n}\max\left(IoU\left(gt{\text{box}}_{p},C\right)\right). \end{array}$$

We set the number of clusters (K) to 4 after performing experiments, as shown in Table 1. We performed four sets of experiments, wherein the number of cluster centers (K) was set to 4, 6, 8, and 10. We found that K is proportional to Avg IoU. Moreover, it is well known that K is proportional to the detection time. Furthermore, we tested the Avg IoU (without the K-means algorithm) with the coordinates of the six cluster centers set by human experience. We found that the Avg IoU was 63.2%. Therefore, to improve the efficiency of the model, we chose K to be 4.

## 5. DMRF-CNN

**Figure 5:**
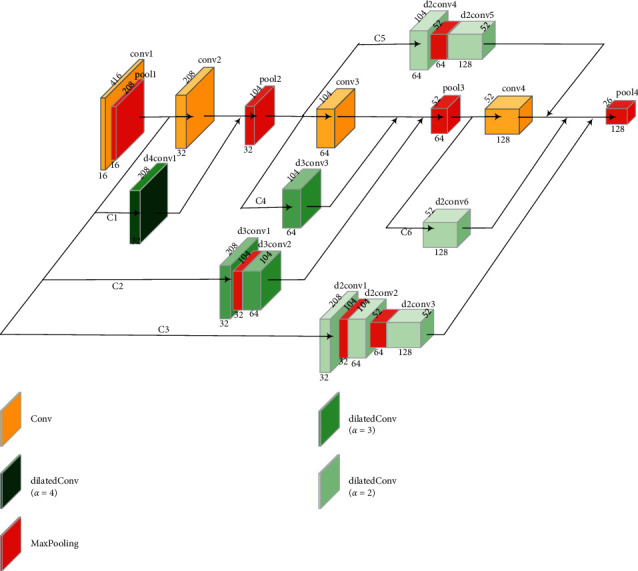
Structure and detailed parameters of DMRF-CNN.

Clinically, the edge and the texture features of thyroid nodules are the critical features for recognition of the benign or malignant type of the nodules. To extract the edge and the texture features, we present DMRF-CNN that uses dilated convolution [39] and cross-layer connections. The details of DMRF-CNN are provided in Figure 5. The label, d-x-conv-y, indicates dilated convolution, where *x* is the dilation rate and *y* denotes the convolution layer. Conv represents a traditional convolution. Cx represents the name of a connection. Different colors represent the feature maps with different operations.

**Figure 6:**
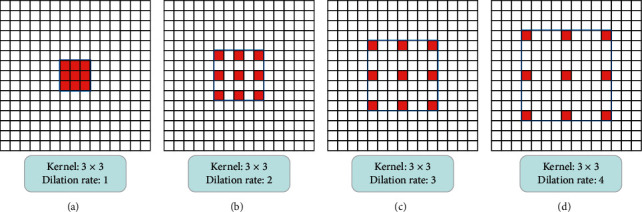
Different kernel sizes corresponding to different dilation rates (here, the 3 × 3 convolution kernel size is considered as an example): (a) the dilation rate is 1(conventional convolution kernel); (b) the dilation rate is 2; (c) the dilation rate is 3; (d) the dilation rate is 4.

We combine the traditional convolution (dilation rate of 1) with dilated convolution in DMRF-CNN. Dilated convolution can enlarge the receptive fields with various dilation rates. Different dilation rates correspond to different convolution kernel sizes. We use the batch normalization and the leaky rectified linear unit (Leaky ReLU) layers after the dilated convolution layer to avoid gradient disappearance. We term the aforementioned dilated operation as the dilatedConv block. In the experiments in this study, we used three dilation rates: 4, 3, and 2, as shown in Figure 6. Furthermore, we use high dilation rate in the shallow layers and low in deep layers. The value of the parameter of the Leaky ReLU is 0.1.

In this work, dense connections are deployed to improve the information transmission between different layers. We use the add operation that adds feature maps to connect two feature maps. For example, the maps: d4conv1 and conv2 are connected by an add operation. We perform max pooling down-sampling to ensure that the feature maps have the same size before the add operation. The dimensions of the feature map after pool4 are 26 × 26 × 128.

### 5.1. Detection and Recognition

Based on the feature maps encoded by the DMRF-CNN, we achieve the detection and classification of thyroid nodules. In this work, two scales are considered to recognize the nodules of different sizes. For each scale, we set two bounding boxes. A tuple comprising four items, i.e., (*x*, *y*, *w*, and *h*) is used to present a bounding box. Here, *x* and *y* denote the relative coordinates of the center of the bounding box. Furthermore, *w*and *h* denote the width and height of the box, respectively. We use confidence to evaluate the accuracy of detection, which is computed by as

(4) $$\begin{array}{c} \text{Conf}=\partial \cdot \text{IoU}\left(T,P\right). \end{array}$$

IoU(*T*, *P*) denotes the IoU of the ground truth and the prediction bounding box. Each thyroid nodule original image is divided into different scale grid cells. In this study, we obtain two scales division: by 13 × 13 and 26 × 26. Two anchor boxes are set for each scale. For each grid cell, we predict two bounding boxes. ∂ has two values: 1 and 0. If the center points of the ground truths are in the current grid cell, ∂ = 1; otherwise, ∂ = 0.

**Figure 7:**
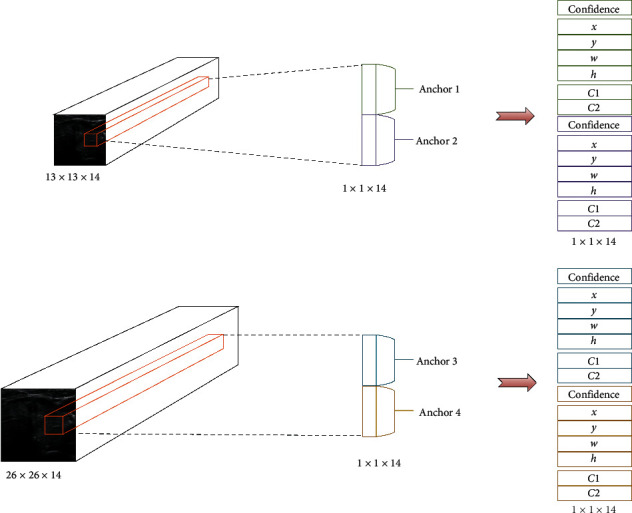
Channel design of detection layers.

In this work, we use a tensor comprising *x*, *y*, *w*, *h*, confidence, and the classification probability for each bounding box, as shown in Figure 7. For each scale recognition, we form a 1 × 1 × 14 evaluation. Here, C1 and C2 denote the prediction probability for the benign and malignant states, respectively. Based on the prediction bounding boxes, we use the nonmaximum suppression (NMS) algorithm [40] to ensure that one thyroid nodule has only one bounding box.

The loss function of YOLOv3-DMRF has three parts: classification loss, localization loss, and confidence loss, which are shown by equation (5). In equation (5), *s*^2^ denotes the number of divided grid cells with B bounding boxes for each grid cell. *μ*_weight_ and *μ*_noboj_ denote the contribution rate. *μ*_weight_ can increase the difference in the localization loss, whereas *μ*_noboj_ can decrease the loss from the confidence predictions for the bounding boxes that do not contain thyroid nodules. In the experiment, we set *μ*_weight_ = 5 and *μ*_noboj_  = 0.5. Conf denotes the confidence, as shown in equation (4). Furthermore, *p*(*c*) refers to the classification prediction. *τ*_*i*_^obj^ is 1 if object appears in the *i*^th^ grid cell; otherwise, the result is 0. The value of *τ*_*ij*_^obj^is 1 if the *j*^th^ bounding box predictor is in *i*^th^ grid cell; otherwise, 0:

(5) Loss=μweight∑i=0s2∑j=0Bτijobjxi−x^i2+yi−y^i2+ μweight∑i=0s2∑j=0Bτijobjwi−w^i2+hi−h^i2+ ∑i=0s2∑j=0BτijobjConfi−Conf^i2  + μnoobj∑i=0s2∑j=0BτijnoobjConfi−Conf^i2+ ∑i=0s2τiobj∑c∈classespic−p^ic2.

## 6. Results and Discussion

### 6.1. Experimental Setup

#### 6.1.1. Datasets and Evaluation Metrics

The dataset used in this study was obtained from 240 patients with 699 ultrasound images of thyroid nodules, which were followed by FNAB. They were collected from the physical health center of a local 3A hospital. These ultrasound images belong to 34 males and 177 females. In our dataset, each image contains at least one thyroid nodule. There are 360 benign and 486 malignant nodules. A total of 10,485 images were obtained through data augmentation. Details of the training and test datasets are provided in Table 2.

In the experiment, we used the metrics: average precision (AP) and mean average precision (mAP) to evaluate the detection and recognition of thyroid nodules. In addition, we use f1 score, recall, accuracy, and precision to evaluate the classification performance of thyroid nodules. They are calculated as follows:

(6) $$\begin{array}{c} \text{AP}=\sum_{n=1}^{N}P\left(n\right)\Delta r\left(n\right), \end{array}$$

(7) $$\begin{array}{c} \text{mAP}=\frac{1}{M}\sum_{m\in M}\text{AP}\left(m\right), \end{array}$$

where *N* represents the total number of images in the test sets, *P*(*n*) is the value of precision, and ∆*r*(*n*) denotes the recall value. The metric, mAP, represents the average of multiple categories of APs, and *M* is the number of classifications:

(8) $$\begin{array}{c} \text{precision}=\frac{\text{TP}}{\text{TP}+\text{FP}}\;, \end{array}$$

(9) $$\begin{array}{c} \text{accuracy}=\frac{\text{TP}+TN}{\text{TP}+\text{FP}+\text{TN}+\text{FN}}, \end{array}$$

(10) $$\begin{array}{c} \text{recall}=\frac{\text{TP}}{\text{TP}+\text{FN}}\;, \end{array}$$

(11) $$\begin{array}{c} f1\text{score}=\frac{2*\text{precision}*\text{recall}}{\text{precision}+\text{recall}}, \end{array}$$

where TP, TN, FP, and FN represent true positives (TP), true negatives (TN), false positives (FP), and true negatives (FN), respectively.

#### 6.1.2. Parameter Setup

The experiments in this study are based on the improved YOLOv3 object detection framework. We used a random gradient descent (SGD) for 60 K iteration training with an initial learning rate of 0.01 and a batch size of 16 images. At the iterations of 40 K and 50 K, the learning rate is reduced by 10 times. To demonstrate the efficiency of YOLOv3-DMRF, we compared it with some state-of-the-art networks based on YOLOv3 such as YOLOv3-tiny, YOLOv3-spp, YOLOv3-320, YOLOv3-416, and YOLOv3-608 on our and an open-access dataset. YOLOv3-spp denotes spatial pyramid pooling (SPP) based on YOLOv3. YOLOv3-320, YOLOv3-416, and YOLOv3-608 represent the different input shapes for DarkNet based on YOLOv3. Furthermore, we also compared the effects of different layer connections and different dilation rates on the precision of the framework. Moreover, we compared the feature maps of different dilation rates to obtain the results of different dilation rates. And we compared the proposed DMRF-CNN with some state-of-the-art CNN models to better demonstrate the performance on the same metrics.

### 6.2. Results and Analysis

#### 6.2.1. Evaluation of Layer Connection and Different Dilation Rates

We compare different dilation rates, as shown in Table 3. In this experiment, we set each convolution as a dilated convolution, and the dilation rates are listed in Table 3. It is evident that, compared with traditional convolution, the mAP improves on using dilated convolution. The mAP shows the best value when the dilation rate is 2.

In this work, we evaluate the layer connection on the dilated convolution (dilation rate is 1) operations. Here, six different connections are used as shown in Table 4. The mean values of Cx are shown in Figure 5. As seen in Table 4, six group experiments are developed to evaluate the layer connection. The results show that the optimal mAP is achieved when all six connection methods are used.

According to Table 4, experiment VI reaches the best performance. Thus, we combine this full layer connection with different dilation rates in follow-up experiments. Different dilation rates will correspond to different results on the layer connection. And the specific results are shown in Table 5. Specifically, the experiments I, II, and III only combine two different dilation rates, and the mAP increases by 2.89%, 1.53%, and 1.99%, respectively, compared to the 85.43% (Table 4, VI). These results revalidate that adding dilated convolution can increase the performance to some extent. In other experiments, we try to fuse dilation rates of no less than three various combinations. It is clear that VII fusion with the four different dilation ratios outperforms other fusions. We employ the structure of the VII model to construct the YOLOv3-DMRF framework.

**Figure 8:**
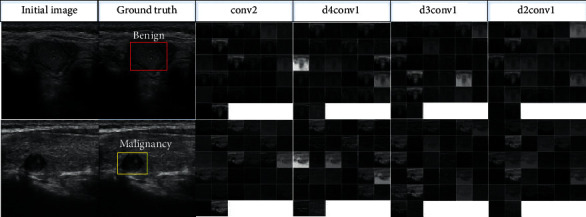
Comparisons between traditional and dilated convolutions.

Figure 8 presents the comparisons between the traditional (dilation rate = 1) and the dilated convolutions (dilation rate = 4, 3, 2). It presents the feature maps generated by convolution kernels with different dilation rates. Here, conv2 is the traditional convolution, and d4conv1, d3conv1, and d2conv1 are the dilated convolutions for rates 4, 3, and 2, respectively. It is evident that the dilated convolutions outperform the traditional one on the extraction of texture and edge features.

#### 6.2.2. Evaluation of DMRF-CNN on Our Dataset

**Figure 9:**
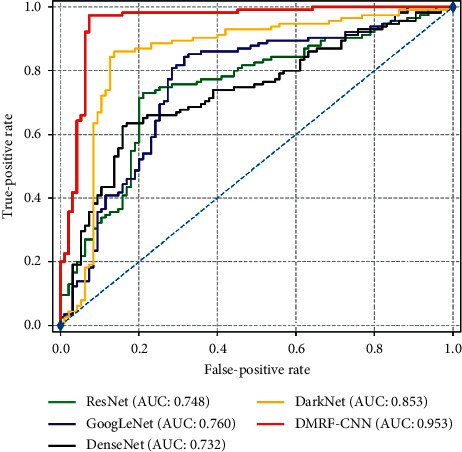
The ROC curve of DMRF-CNN and some state-of-the-art networks.

To better validate the feasibility of the designed DMRF-CNN architecture, we also retain some state-of-the-art networks on our thyroid dataset. For the dataset, we split it refer to above Table 2. Before training, we crop and resize the thyroid nodules of ultrasound images to the same size (240 ∗ 240). To quickly attain the convergence of every model, we utilize SGD as the optimizer and employ cross-entropy as the loss function.  Table 6 describes specific comparisons between DMRF-CNN and these structures on four metrics the classification accuracy, f1 score, precision, and recall. DMRF-CNN achieves the best performance on all four metrics, especially for the recall (97.39%). For other models, we conclude that DarkNet shows stability and reaches the same level on these metrics. The precision on DarkNet is 88.39%, ranking second behind the DMRF-CNN. However, the densely connected convolutional networks (DenseNet) [41] obtain the disappointing results on all metrics except the precision, showing the fluctuation. Meanwhile, Figure 9 illustrates the corresponding ROC curves. Each color represents each network architecture. It is clear that our proposed method DMRF-CNN gets the best performance and the AUC is 95.3%. The AUC value of DarkNet is 0.853, which is only behind DMRF-CNN. This again demonstrates the stability of the DarkNet, even if it cannot keep up with the AUC of DMRF CNN. Similarly, the worst value is still DenseNet, and our method ranks 22.1 percentage points higher in the AUC than it.

#### 6.2.3. Evaluation of Different Models on Our Dataset

**Figure 10:**
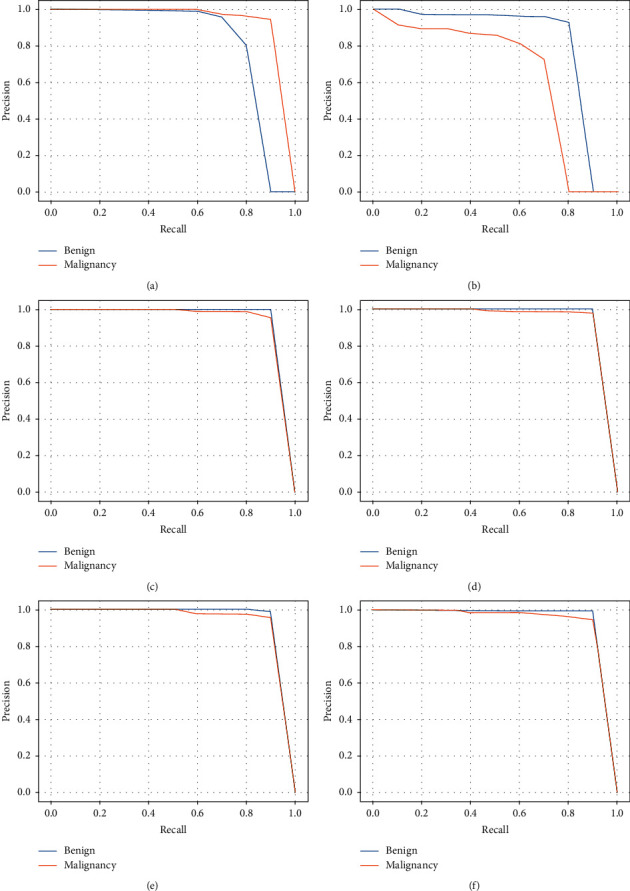
PR curve of different methods: (a) PR curve of YOLOv3-tiny; (b) PR curve of YOLOv3-spp; (c) PR curve of YOLOv3-320; (d) PR curve of YOLOv3-416; (e) PR curve of YOLOv3-608; (f) PR curve of YOLOv3-DMRF.

As shown in Table 7, we compare the mAP for YOLOv3-DMRF with that of the state-of-the-art-models based on YOLOv3. It is seen that YOLOv3-416 achieves mAP of 90.58% and the detection time is 9 s. This could be caused by the model depth of 106. The YOLOv3-DMRF model achieves mAP of 90.05%, and the detection time is very short, i.e., 3.7 s. As shown in Figure 10, the benign and malignant AP values of YOLOv3-320, YOLOv3-416, and YOLOv3-608 achieve AP values for benign and malignant types; however, the cost of detection time is very high than that of YOLOv3-DMRF. Compared to other models, the mAP of YOLOv3-DMRF does not differ much; however, its detection speed is three times greater.

**Figure 11:**
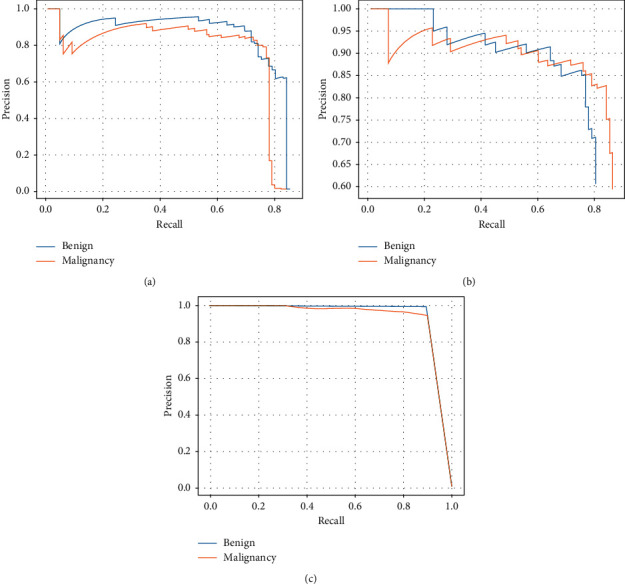
The PR curves of YOLOv3-DMRF and some object detection methods: (a) PR curve of Fast R-CNN; (b) PR curve of SSD; (c) PR curve of YOLOv3-DMRF.

Furthermore, we compare the performance of YOLOv3-DMRF with other object detection algorithms, as shown in Table 8. Through this data analysis, we may draw the conclusion, our proposed method is not only higher in mAP than other object detection frameworks but also better in detection time. Our proposed method has the same AP between benign and malignant; thus, our model is far more stable than other object detection frameworks that owe high AP on malignant because the detection algorithms cannot detect small nodules that are usually diagnosed with benign nodules. In addition, inspired by the one-stage structure of SSD and YOLOv3-DMRF, their detection time outperforms the Fast R-CNN. In addition, we draw the PR curve of three methods in Figure 11. It is seen that our proposed methods outperform other methods in the AP number on different classes. The low diversity of YOLOv3-DMRF between the two categories cannot be ignored.

#### 6.2.4. Evaluation on Open-Access Datasets

In this work, we evaluate the universality of YOLOv3-DMRF on open datasets [32]. The open-access dataset includes 299 patients, of whom 270 are women and 29 are men. We treat the labels 4c and 5 in this open-access dataset as the malignant nodules while others as benign ones. We acquired 111 malignant and 41 benign thyroid nodules. As shown in Table 9, our network outperforms other state-of-the-art networks based on YOLOv3.

Furthermore, we evaluate the mAP with different object detection methods on the open-access dataset. As shown in Table 10, we can see that the detection time and mAP of our methods outperform other methods. At the same time, the AP number of two classes is stable, and the AP number is 92.68% and 97.59%, respectively.

#### 6.2.5. Visualization

**Figure 12:**
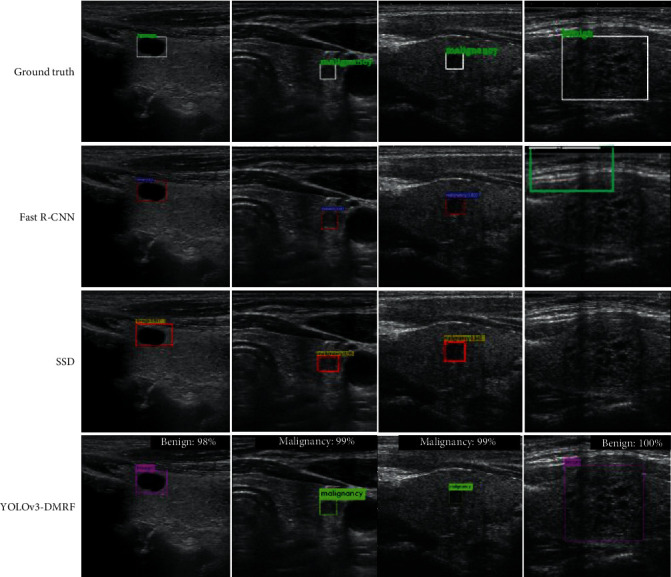
Detection using our method and other object detection methods. The first and second columns are test images form our dataset, and the last two columns are test images from open dataset.

In this work, we present four images (two images from our dataset and two images from the open dataset) of thyroid nodules. The results of the recognition are shown in Figure 12. In the first and second columns are test images from our dataset, and we can see that the bounding box of Fast R-CNN method outperforms the SSD method, but the classification and detection time of SSD outperforms Fast R-CNN. The bounding box of the YOLOv3-DMRF method is not only close to the ground truth but also the classification outperforms Fast R-CNN and SSD methods. Especially, the accuracy of our method is 6.3 percentage points higher than Fast R-CNN for benign nodules because our proposed method used a multidetection layer. In the public dataset, we can see that the Fast R-CNN and SSD methods cannot detect the benign nodule, but YOLOv3-DMRF can detect this nodule and the accuracy of classification is 100 percent.

## 7. Conclusions

In this paper, we proposed YOLOv3-DMRF, based on YOLOv3, to detect and recognize thyroid nodules efficiently. We presented DMRF-CNN to extract the edge and the texture features of thyroid nodules. Especially, we compared some state-of-the-art CNN models. The results showed that DMRF-CNN has a good stability, and the AUC number is 95.3% in our dataset. We used a multiscale detection layer to recognize different sizes of the nodules. The experimental results showed that YOLOv3-DMRF outperforms other models on performance and detection time, and mAP was 90.05% on our dataset. Moreover, we evaluated YOLOv3-DMRF on an open-access dataset, where it achieved good mAP and detection time. The mAP was 95.23%, and the detection time was 2.2 s, which are very good compared to other models. In future, we will continue to collect ultrasound images of thyroid nodules to improve the mAP of our method. Additionally, we plan to further classify the malignant nodules by using ultrasound images.

## Figures and Tables

**Algorithm 1:**
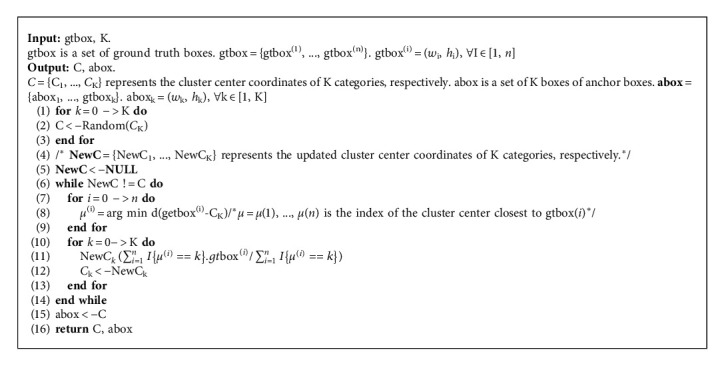
K-means get anchor box.

**Table 1:** Avg IoU for K-means.

K	4	6	8	10
Avg IoU (%)	67.82	71.02	74.69	76.93

**Table 2:** Details of training and test datasets.

	Training dataset	Test dataset	Total
Original datasets	490	209	699
Augmented datasets	10276	209	10485

**Table 3:** mAP of different dilation rates.

Dilation rate	1	2	3	4
mAP (%)	85.06	**88.95**	87.61	84.68

**Table 4:** mAP of different connections.

Name	C1	C2	C3	C4	C5	C6	mAP (%)
I							85.06
II	^*∗*^	^*∗*^	^*∗*^				85.37
III				^*∗*^	^*∗*^		84.72
IV	^*∗*^	^*∗*^	^*∗*^	^*∗*^	^*∗*^		84.99
V	^*∗*^	^*∗*^	^*∗*^			^*∗*^	85.01
**VI**	^*∗*^	^*∗*^	^*∗*^	^*∗*^	^*∗*^	^*∗*^	**85.43**

**Table 5:** mAP of fusion of different dilation rates.

Name	Dilation rate = 1	Dilation rate = 2	Dilation rate = 3	Dilation rate = 4	MAP (%)
I	^*∗*^	^*∗*^			88.32
II	^*∗*^		^*∗*^		86.96
III	^*∗*^			^*∗*^	87.42
IV	^*∗*^	^*∗*^	^*∗*^		88.54
V	^*∗*^		^*∗*^	^*∗*^	87.91
VI	^*∗*^	^*∗*^		^*∗*^	89.54
VII	^*∗*^	^*∗*^	^*∗*^	^*∗*^	**90.05**

**Table 6:** The metrics of DMRF-CNN and some state-of-the-art networks.

Model	Accuracy	f1 score	Precision	Recall
ResNet [19]	75.24	75.93	81.19	71.30
GoogLeNet [18]	77.14	80.33	75.97	85.22
DarkNet [28]	86.19	87.22	88.39	86.09
DenseNet [41]	72.38	71.29	82.76	62.61
**DMRF-CNN**	**95.24**	**95.73**	**94.12**	**97.39**

**Table 7:** mAP of different networks on our dataset.

Model	Benign (AP) (%)	Malignant (AP) (%)	Detection time in all test images (s)	mAP on our dataset (%)
YOLOv3-tiny	79.50	89.86	2	84.68
YOLOv3-spp	79.43	63.30	11.3	71.36
YOLOv3-320	90.91	90.18	9.1	90.54
YOLOv3-416	90.91	90.26	9.4	90.58
YOLOv3-608	90.79	89.86	9.8	90.32
**YOLOv3-DMRF**	**90.66**	**89.43**	**3.7**	**90.05**

**Table 8:** Evaluation of YOLOv3-DMRF and other object detection on our dataset.

Model	Benign (AP) (%)	Malignant (AP) (%)	Detection time in all test images (s)	mAP on our dataset (%)
Fast R-CNN [26]	75.08	66.36	239.54	70.72
SSD [25]	75.06	79.47	22.4	77.27
**YOLOv3-DMRF**	**90.66**	**89.43**	**3.7**	**90.05**

**Table 9:** mAP of different networks for an open-access dataset.

Model	Benign (AP) (%)	Malignant (AP) (%)	Detection time in all test images (s)	mAP (%)
YOLOv3-tiny	72.73	80.72	0.9	76.72
YOLOv3-spp	49.59	57.88	9	53.73
YOLOv3-320	72.73	89.87	4.5	81.3
YOLOv3-416	72.73	98.04	4.9	85.38
YOLOv3-608	79.97	89.83	8	84.85
**YOLOv3-DMRF**	**92.68**	**97.59**	**2.2**	**95.23**

**Table 10:** Evaluation of YOLOv3-DMRF and other object detection on open-access dataset.

Model	Benign (AP) (%)	Malignant (AP) (%)	Detection time in all test images (s)	mAP (%)
Fast R-CNN [26]	59.29	75.75	127.6	67.52
SSD [25]	57.55	89.33	10.21	73.44
**YOLOv3-DMRF**	**92.68**	**97.59**	**2.2**	**95.23**

## Data Availability

The collected data used to support the findings of this study are restricted by the Department of Health Management, Henan Provincial People's Hospital in order to protect patient privacy. The collected data are available from Runzhi Li via rzli@ha.edu.cn for researchers who meet the criteria for access to confidential data. The open-access data used to support the findings of this study are included within the article.
